# The Pore-Forming Hemolysin BL Enterotoxin from *Bacillus cereus*: Subunit Interactions in Cell-Free Systems

**DOI:** 10.3390/toxins13110807

**Published:** 2021-11-15

**Authors:** Franziska Ramm, Marlitt Stech, Anne Zemella, Hendrik Frentzel, Stefan Kubick

**Affiliations:** 1 Fraunhofer Institute for Cell Therapy and Immunology (IZI), Branch Bioanalytics and Bioprocesses (IZI-BB), Am Mühlenberg 13, 14476 Potsdam, Germany; franziska.ramm@izi-bb.fraunhofer.de (F.R.); marlitt.stech@izi-bb.fraunhofer.de (M.S.); anne.zemella@izi-bb.fraunhofer.de (A.Z.); 2 Institute of Chemistry and Biochemistry, Freie Universität Berlin, Thielallee 63, 14195 Berlin, Germany; 3 Department of Biological Safety, German Federal Institute for Risk Assessment, Max-Dohrn-Str. 8-10, 10589 Berlin, Germany; hendrik.frentzel@bfr.bund.de; 4 Faculty of Health Sciences, Joint Faculty of the Brandenburg University of Technology Cottbus–Senftenberg, Brandenburg Medical School Theodor Fontane and the University of Potsdam, 14476 Potsdam, Germany

**Keywords:** eukaryotic cell-free system, tripartite enterotoxin, hemolytic enterotoxin, hemolysin BL, *Bacillus cereus*, membrane perforation

## Abstract

The tripartite enterotoxin Hemolysin BL (Hbl) has been widely characterized as a hemolytic and cytotoxic virulence factor involved in foodborne diarrheal illness caused by *Bacillus cereus*. Previous studies have described the formation of the Hbl complex and aimed to identify the toxin’s mode of action. In this study, we analyzed the assembly of Hbl out of its three individual subunits L_1_, L_2_ and B in a soluble as well as a putative membrane bound composition using a Chinese hamster ovary (CHO) cell-free system. Subunits were either coexpressed or synthesized individually in separate cell-free reactions and mixed together afterwards. Hemolytic activity of cell-free synthesized subunits was demonstrated on 5% sheep blood agar and identified both synthesis procedures, coexpression as well as individual synthesis of each subunit, as functional for the synthesis of an active Hbl complex. Hbl’s ability to perforate cell membranes was evaluated using a propidium iodide uptake assay. These data suggested that coexpressed Hbl subunits augmented cytotoxic activity with increasing concentrations. Further, a pre-pore-complex of L_1_-L_2_ showed cytotoxic effects suggesting the possibility of an interaction between the cell membrane and the pre-pore-complex. Overall, this study shows that cell-free protein synthesis is a fast and efficient way to study the assembly of multiple protein subunits in soluble as well as vesicular fractions.

## 1. Introduction

*Bacillus cereus* produces various virulence factors including phospholipases, hemolysins and enterotoxins [1,2]. One of the enterotoxins considered as a causative factor for the *B. cereus* associated diarrheal disease is the multicomponent toxin named Hemolysin BL (Hbl). Early studies have identified three individual antigens that were described as a B binding component and two lytic components, namely L_1_ and L_2_ [3,4]. These subunits are encoded in the *hblCDA* operon where *hblC* relates to the L_2_, *hblD* to the L_1_ and *hblA* to the B Component. Further studies revealed a hemolytic activity when all three subunits were present. It was concluded that the resulting hemolysis is particularly characterized by a ring-shaped zone offsite of the center of the applied toxin. This phenomenon was later explained by a diffusion of the subunits out of an area of L_1_ excess which inhibits the hemolytic activity [5,6,7]. Even though Hbl does not only show lytic activity but also enterotoxic activity in rabbit ileal loops [7,8], the mode of action was mostly studied using hemolytic systems. Initial studies suggested that all subunits can independently bind to erythrocytes and the lysis is thereby caused by a “membrane attack complex” with the formation of a transmembrane pore of 1.2 nm in diameter [6]. Later studies indicated a sequential binding order of the B Component followed by the lytic component L_1_ and L_2_ at last [9]. The formation of soluble B and L_1_ as well as L_1_ and L_2_ complexes could be verified by surface plasmon resonance spectroscopy (SPR), dot blot analysis and enzyme immuno assays (EIA) [10]. An oligomerization of the B Component into hepta- or even octamers was suggested [11], but the complete mode of action of the complex formation still has to be further elucidated. The rapid kinetics and the contribution of each individual subunit to the activity of the whole complex were vastly analyzed, indicating that an excess of the binding Component B led to an accelerated pore-formation while an excess of the lytic components, in particular L_1_, delayed this response [12]. Additional studies have shown an increased effect of the Hbl tripartite enterotoxin when sphingomyelinases and phosphatidylcholine phospholipases C from *Bacillus cereus* were present [13]. It is rather difficult to isolate specific toxins from bacterial strains producing a variety of toxins. Further, the recombinant production and purification of bacterial toxins can be very challenging [10]. Therefore, the synthesis of individual toxin subunits and their detailed analysis within the whole pore-forming complex is of upmost interest. The synthesis of Hbl in prokaryotic systems has been successfully established in previous studies, but protein purification was necessary [9]. The addition of tags for purification could alter the folding behavior of the protein itself, which could result in inactive proteins or multicomponent proteins might not assemble. Cell-free protein synthesis (CFPS) is an alternative as proteins synthesized in a eukaryotic system can be directly used for functional assessments without the need for purification. CFPS is a time- and cost-efficient way to produce single proteins, glycoproteins, protein-complexes as well as “difficult-to express” proteins such as membrane proteins and toxic proteins in a well-defined model system [14,15,16,17,18,19,20,21]. Single proteins can be synthesized and functionally characterized individually as well as in a mixture of different proteins. Limiting factors such as the cell’s viability or membrane barriers can be neglected as a crude cell lysate instead of intact cells is used [19,22]. The open system allows for the supplementation of variable components required for the protein’s need or for further analyses. Therefore, the supplementation of radioactively labeled amino acids allows for the precise protein quantification without the need of any specific antibodies. CFPS is not limited to the use of radioactivity but can fluorescently label the protein of interest site-specifically [14] or randomly [23]. In this study, we present cell-free protein synthesis as a tool to rapidly and efficiently study multicomponent toxins and analyze the complex formation out of single subunits in soluble and in a putative membrane bound manner. Here, we examine the Hbl tripartite enterotoxin from *Bacillus cereus*, which has not been synthesized in a eukaryotic expression system before. Our cell-free system enables the direct application of the toxin, and toxin subunits in cell-based toxicity assays without prior purification steps as no cytotoxic compounds such as endotoxins are present in eukaryotic cell-free systems. CFPS facilitates the application of multiple synthesis conditions offering the possibility to study the single Hbl subunits as well as their co-expressed complex form. Adding the individual subunits in a sequential manner to the synthesis reaction qualifies the cell-free system for the detailed study of subunit interactions.

## 2. Results

The characterization of bacterial enterotoxins is generally based on the use of culture supernatant, which contains various bacterial proteins, and therefore requires intense purification steps. In this context, Hbl enterotoxins have been isolated, purified and characterized extensively in the 1990s [4,5,7] while latest research has focused on the complex formation and binding properties of Hbl using deletion mutants, recombinant toxins and/or antibody neutralization [9,10,12]. Nonetheless, the precise mode of action has yet to be further clarified. Here we report the individual synthesis of all Hbl enterotoxin subunits using eukaryotic cell-free systems based on CHO-lysates. In addition, we demonstrate their cell-free coexpression. Following their synthesis, we characterize soluble proteins as well as putatively membrane bound enterotoxin without the necessity of time-consuming purification steps.

### 2.1. Establishing Cell-Free Synthesis of Hemolysin BL

**Figure 1:**
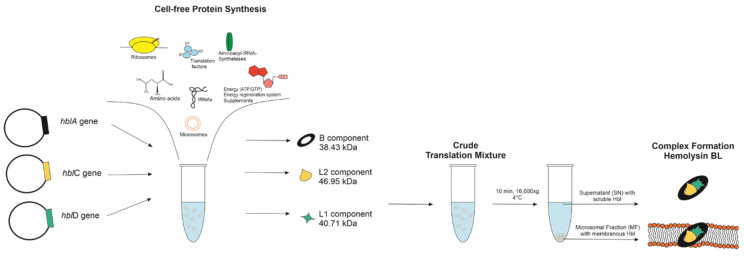
Schematic illustration of the cell-free protein synthesis of the Hbl complex.

As mentioned above, prior work has shown that Hbl subunits interact with one another and form a tripartite complex as well as pre-pore complexes [10]. Our eukaryotic cell-free systems offer the possibility to add any number of subunit encoding genes and supplements to the cell-free synthesis as a prerequisite to study the embedding of the Hbl enterotoxin into endogenous membranous structures present in our lysates. Such microsomes were derived from the endoplasmic reticulum, retaining their activity throughout the entire lysate preparation procedure [24]. In order to establish the synthesis of the Hbl tripartite enterotoxin, all subunits were produced individually in separate reactions. As an experimental alternative, two subunits as well as all three subunits were synthesized simultaneously within the same cell-free reaction compartment (coexpression). After the synthesis, the crude translation mixture (TM) was fractionated into the soluble fraction (supernatant, SN) and the microsomal fraction (MF) as represented in Figure 1.

**Figure 2:**
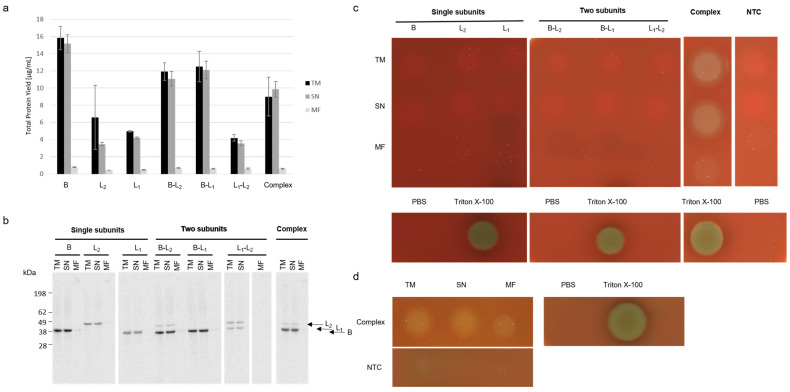
Cell-free synthesis of Hbl. Hbl subunits were synthesized in CHO lysates either separately or in a coexpression of either two or three subunits. (**a**) Quantitative analysis of cell-free synthesized Hbl and subunits as performed by liquid scintillation counting. Standard deviations were calculated from triplicate analysis. (**b**) Autoradiograph showing ^14^C-leucine labeled Hbl single subunits and coexpressed subunits when synthesized using a molar plasmid ratio of 1:1 for two subunits or a ratio of 1:1:1 for tripartite coexpression. (**c**) Hemolytic activity of 10 µL of the single subunits, two coexpressed subunits and the Hbl complex was assessed on 5% sheep blood agar plates. (**d**) Hemolytic activity on 5% sheep blood agar plates of Hbl complex at a defined concentration of 1 µg/mL for TM, SN and MF fractions. A no template control (NTC) and PBS were used as a negative control. Triton-X 100 was used as a positive control.

Quantitative analysis showed that the B Component was synthesized in higher concentrations compared to the L_1_ and L_2_ components in individual and coexpressed synthesis. In general, the quantitative analysis showed higher overall amounts of soluble protein compared to the MF (Figure 2a). Qualitative analysis via autoradiography depicted precise protein bands for single subunits in the TM and SN while less intense or no bands could be detected for B and L_2_ or L_1_ components in the MF, indicating that only a small proportion of the subunits is bound to microsomal membranes (Figure 2). Coexpressed subunits showed intense protein bands at around 38–40 kDa representing both the B and L_1_ component at a similar molecular weight whenever both proteins were synthesized (Figure 2b). At last, hemolytic activity of single subunits and two as well as three coexpressed subunits was assessed on 5% sheep blood agar plates. Single subunits as well as two coexpressed subunits showed no hemolytic activity in neither fraction (Figure 2c, uncropped plate Figure S1). Nonetheless, when proteins were spotted in close proximity to each other on one agar plate, crescent shaped areas occurred at zones between spotted L_1_ component and coexpressed B-L_2_ components (Figure S1). These data correlate to previous findings, indicating a diffusion of the subunits within the blood agar plate, causing the lysis of erythrocytes upon subunit interaction [5]. Defined hemolytic activity of the three coexpressed subunits was shown in the TM and SN fraction. The MF fraction showed hemolytic activity but not as intense as for the soluble protein (Figure 2c, uncropped plate Figure S1). To compare the hemolytic activity of the individual fractions after coexpression, each fraction was diluted to the same concentration and spotted onto the blood agar plate. These data depicted the reduced hemolytic activity of the MF fraction in comparison to the soluble fraction (Figure 2d, uncropped plate Figure S2). In addition to the previous data, a concentration dependent size of the hemolytic area could be observed when samples were spotted onto the 5% sheep blood agar plate at different concentrations (Figure S2).

### 2.2. Subunit Interactions in Soluble and Microsomal Fractions

**Figure 3:**
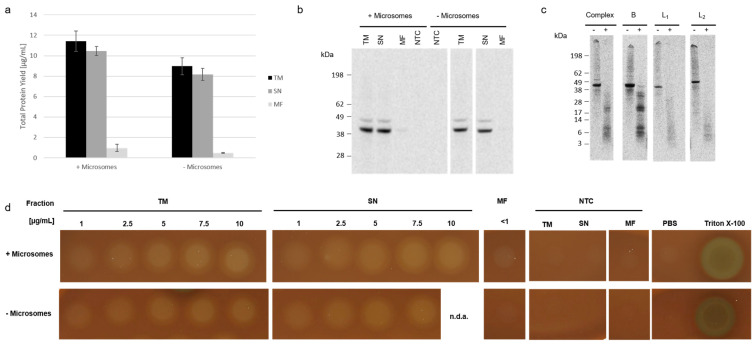
Cell-free synthesis of Hbl examined in lysates with and without microsomes. Hbl subunits B, L_2_ and L_1_ were synthesized in CHO lysates in a coexpression combining all three subunits. (**a**) Quantitative analysis using liquid scintillation counting. Standard deviations were calculated from triplicate analysis. (**b**) Autoradiograph showing ^14^C-leucine labeled coexpressed Hbl subunits when synthesized using molar plasmid concentrations in a 1:1:1 ratio. (**c**) Autoradiograph showing ^14^C-leucine labeled Hbl single subunits and coexpressed subunits when synthesized using a molar plasmid ratio of 1:1 for two subunits or a ratio of 1:1:1 for tripartite coexpression before (−) and after (+) a proteinase K digestion. (**d**) Hemolytic activity of the Hbl complex was assessed on 5% sheep blood agar plates. A total of 10 µL of increasing concentrations [1–10 µg/mL] were spotted onto the blood agar plate. The TM, SN and MF were analysed. The amount of 10 µg/mL for SN fraction in a microsome depleted lysate could not be reached (=no data available, n.d.a.).

The three subunits of Hbl were coexpressed in a CHO lysate containing microsomal vesicles and in a CHO lysate depleted from microsomes by centrifugation, respectively. In general, the synthesis in the presence of microsomes resulted in total protein yields above 10 µg/mL while the absence of microsomes led to slightly reduced protein yields below 10 µg/mL (Figure 3a), thus indicating that the cell-free synthesis is more efficient in lysates containing microsomes. Qualitative analysis depicted no differences when using a system with and without microsomes in the TM and SN fraction. As expected, no protein band could be detected in the MF in a microsome depleted system. Again, only a slight protein band was visible in the MF in a microsome containing system (Figure 3b).

As the binding Component B targets cell surfaces, we expected the B Component to target the microsomal vesicles as well. As only slight protein bands were visible in the autoradiographs, the interaction of the Hbl enterotoxin with the microsomes was questioned. To investigate the interaction of the individual subunits with the microsomal vesicles, a proteinase K digestion was performed. In a time-dependent manner, the enzyme digests extracellular as well as extraluminal domains suggesting that soluble subunits would be digested completely whilst a membrane bound subunit would only be digested partially. As a result, a defined band pattern of protein fragments could be detected in the autoradiograph for the binding Component B as well as coexpressions of the B Component with other subunits (Figure 3c). Three protein fragments were detected in the range of 3–10 kDa, two between 20–25 kDa and two between 32–35 kDa. The L_1_ and L_2_ subunits did not display this defined band pattern indicating a complete digestion of these subunits and suggesting no interaction with the microsomes (Figure 3c). Coexpressions of two subunits showed similar band patterns when the B Component was present (Figure S3). These results further suggest a binding of the B Component and potential Hbl enterotoxin to microsomal membranes facilitated by the B Component. After proving that Hbl interacts with the microsomes, the individual fractions synthesized with and without microsomes were spotted onto 5% sheep blood agar plates to assess Hbl’s lytic activity. Both, Hbl complexes from microsome-containing and microsome-depleted lysate, showed hemolytic activity at concentrations in the range of 1 and 10 µg/mL. As expected, the MF fraction was only lytic in the approach using microsomes, but only a slight hemolytic activity could be detected (Figure 3d, uncropped plates in Figure S4).

**Figure 4:**
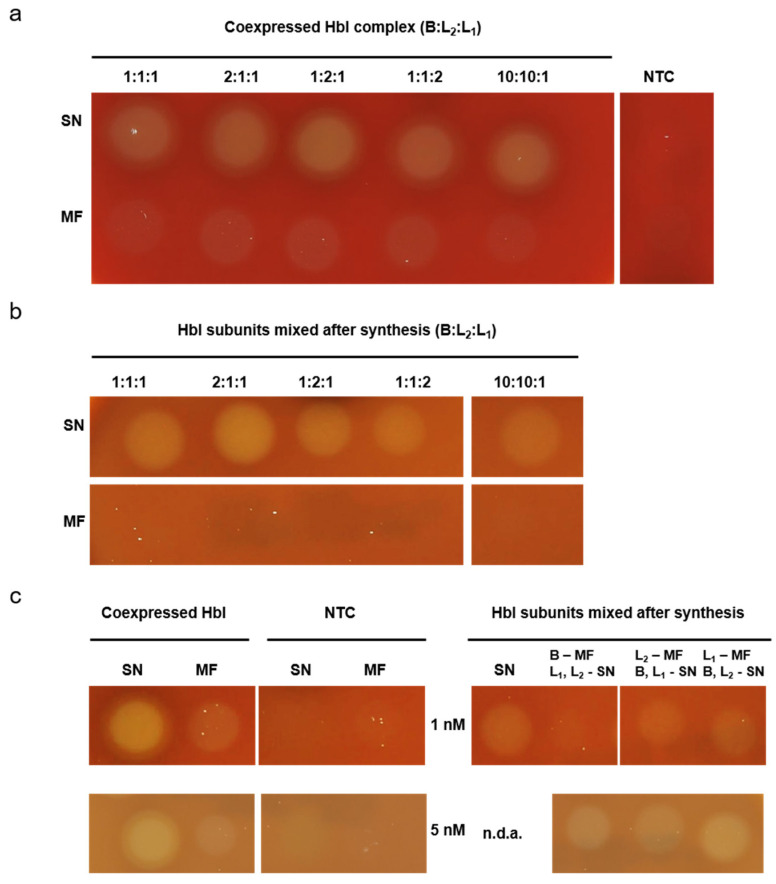
Analysis of Hbl subunit interaction. Hemolytic activity was assessed on 5% sheep blood agar plates. (**a**) Hbl subunits B, L_2_ and L_1_ were coexpressed in CHO lysate using different molar plasmid ratios. (**b**) Hbl subunits were expressed separately in CHO lysate. Subsequently, fractions of each subunit in SN and MF were mixed in different molar protein ratios. (**c**) Hbl subunits were expressed separately in CHO lysate and subsequently two subunits of the SN fraction were mixed with one subunit of the MF: All subunits from SN (SN), B from MF and L_2_ and L_1_ from SN (B-MF), L_2_ from MF and B and L_1_ from SN (L_2_-MF), L_1_ from MF and B and L_2_ from SN (L_1_-MF).

We further aimed to investigate the interaction of the individual subunits with each other in the cell-free system. Therefore, the three different subunits B, L_2_ and L_1_ were coexpressed using defined molar plasmid ratios of 1:1:1, 2:1:1, 1:2:1, 1:1:2 and 10:10:1. In a subsequent step additional ratios were tested, in particular 10:1:1, 1:10:1, 1:1:10, 10:1:10 and 1:10:10, in order to test the effect of 10-fold increases of single subunits on the functionality of the Hbl complex. Following CFPS 10 µL of the soluble as well as the membrane bound fractions were spotted onto a 5% sheep blood agar plate. The ratios were chosen in order to overexpress each individual subunit individually to investigate the influence of the single subunits on the whole Hbl complex. All tested coexpression ratios resulted in an intense lytic activity in the soluble fraction but minimal activity or even no activity was detected in the microsomal fraction (Figure 4a and Figure S5, uncropped plates Figure S6). A decreased hemolytic activity in the soluble fraction could only be observed when overexpressing the L_1_ subunit (Figure S5).

Single subunit interactions were additionally assessed when subunits were individually synthesized in a cell-free manner and combined after the synthesis. Accordingly, molar ratios of protein subunits were chosen corresponding to the molar plasmid ratios used before. Strikingly, hemolytic activity was only observed for the combined individual proteins of the soluble fractions whereas none of the microsomal fractions displayed any hemolytic activity (Figure 4b and Figure S5, uncropped plates Figure S6). Moreover, the B:L_2_:L_1_ ratios of 1:10:1 and 1:1:10 lacked hemolytic activity. In a next step, additional molar protein ratios with a 10-fold increase of individual protein subunits were tested. The results obtained indicated that in the soluble fraction a 10-fold overexpression of L_2_ and L_1_ led to an inhibition of hemolytic activity (Figure S5). In order to evaluate whether the protein concentration in the microsomal fraction was too low to detect the lytic activity on the blood agar plate or a certain subunit inhibited the lytic activity, a putative membrane bound subunit was combined with the two other subunits that appeared to be soluble after cell-free synthesis. Two different concentrations (1 and 5 nM) of Hbl subunits were spotted to identify a concentration dependent effect on the sheep blood agar plates. Hemolytic activity assessment indicated that the tripartite complex was not active when the B Component was administered in a putative membrane bound form at a final concentration of 1 nM but showed lytic activity when administered at 5 nM. Thus, these results showed a concentration dependent effect of the B subunits which was not detected for L_1_ and L_2_ (Figure 4c, uncropped plates Figure S6).

**Figure 5:**
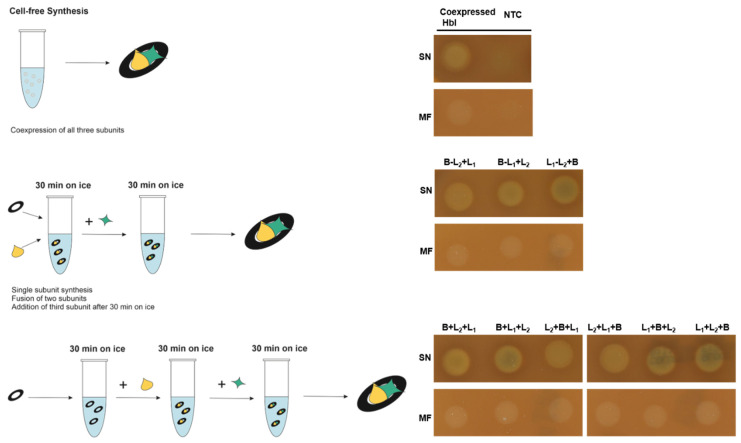
Analysis of Hbl complex formation. Coexpressed Hbl subunits were compared to separately expressed that were fused after the synthesis.

So far, our experiments assessed the interaction of the Hbl subunits by combining all three subunits immediately together after the cell-free synthesis. In order to investigate a putative time dependent influence on the interaction of the subunits we further analyzed the fusion of subunits by directly mixing two subunits and after an initial 30 min incubation step on ice, adding the final subunit. Additionally, adding all three subunits sequentially with a 30 min incubation step in between each subunit supplementation was analyzed. The subunits were mixed in a 1:1:1 protein ratio at a final concentration of 5 and 3 nM for the SN and MF fraction, respectively. All combinations resulted in lytic activity on the 5% sheep blood agar plates indicating a time independent complex formation (Figure 5, uncropped plate Figure S7).

### 2.3. Membrane Perforation of Hemolysin BL

**Figure 6:**
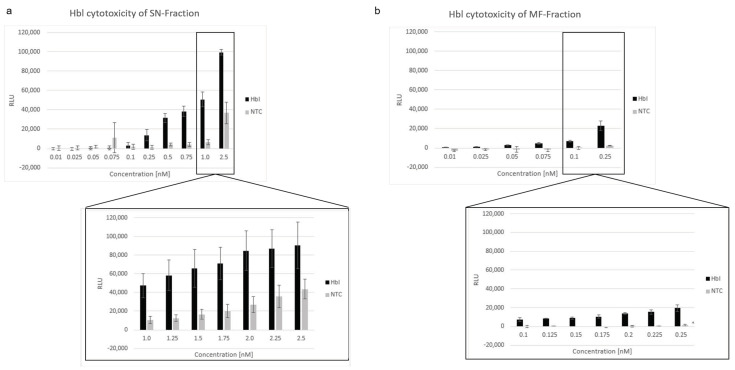
Hbl induced membrane perforation monitored by propidium iodide staining. Cell-free synthesized Hbl was assessed for its cytotoxic effect on CaCo2 cells. Upon Hbl induced membrane rupture, the DNA intercalating agent propidium iodide (PI) enters the cell’s cytoplasm. DNA bound PI was detected at 616 nm using the Mithras LB 943 (Berthold). Hbl tripartite toxin was coexpressed and the SN fraction (**a**) and the MF fraction (**b**) were analyzed. An NTC consisting of a volume equivalent reaction without a coding DNA template was used as a negative control. Hbl protein concentrations ranging from 0.01 nM up to 2.5 nM were tested. Standard deviations were calculated from triplicate samples of three independent experiments (*n* = 9) with the exception of the MF NTC at 0.25 nM as indicated by * (*n* = 5).

As the hemolytic activity in blood agar plates is a qualitative measure, the functional activity of the Hbl complex was further assessed using propidium iodide (PI) uptake studies on CaCo2 cells. The Hbl complex is known to form pores in cell membranes [6]. Therefore, the ability to perforate cell membranes was studied with PI. PI cannot cross the intact cell membrane but when a pore is present within the membrane, PI can enter the cell. It then intercalates with nucleic acids and its emission maximum shifts from 590 nm to 617 nm. Thus, a PI uptake assay reflects the membrane perforation caused by the Hbl complex. Varying concentrations of the coexpressed complex were screened to evaluate the toxic dose on 10,000 CaCo2 cells. The soluble coexpressed subunits induced initial toxic effects at 0.25 nM. The equivalent NTC volume did not result in toxic effects. The toxic effect of the Hbl complex increased with increasing concentrations, which were measured from 0.01 nM up to 2.5 nM (Figure 6a top). Initial toxic effects could be detected at 0.1 nM, but a toxic effect that could precisely be distinguished from the NTC background activity was observed at 0.25 nM. To monitor the concentration range from 1 to 2.5 nM in detail, a second data set was acquired. These data depicted that an increased Hbl concentration showed defined differences in the membrane perforation process even when the concentration was only increased by 0.25 nM (Figure 6a, bottom). To assess the toxicity of the MF fraction a similar concentration range was screened as for the SN fraction. In general, the MF fraction resulted in lower total protein yields compared to the SN fraction. Therefore, the highest concentration tested in the PI assay for the MF fraction was 0.25 nM, the lowest 0.01 nM. This data set clearly depicted initial toxic effects at 0.05 nM (Figure 6b, top). Nonetheless, precise toxic effects were seen at starting concentrations of 0.1 nM. Due to the normalization using the medium control, the NTC showed negative fluorescence counts as the medium treated cells displayed higher background values than the NTC between the 0.01 and 0.075 nM volume equivalents. The concentration range between 0.1 nM and 0.25 nM was monitored in addition. Again, even small increases of the Hbl complex concentration, increased the membrane disruption (Figure 6b, bottom). Analyzing the lysate background from the SN and the MF fraction, the PI uptake assay demonstrated less intense values for the NTC in the MF. The SN showed high NTC values at high concentrations. Overall, these data support the previous findings that both, SN and MF fractions, harbor functionally active Hbl complex.

**Figure 7:**
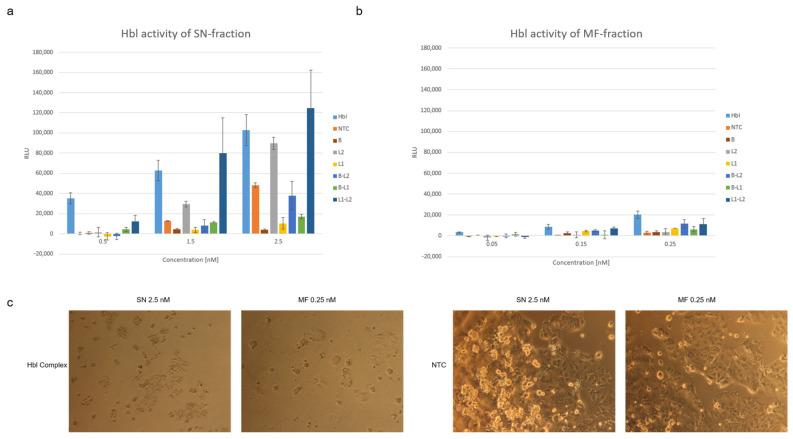
Membrane perforation assay of the individual Hbl subunits. Cell-free synthesized Hbl, the individual subunits as well as two subunits synthesized together were assessed for their cytotoxic effects on CaCo2 cells using the DNA intercalating propidium iodide. The coexpressed Hbl tripartite toxin, the individual subunits and two coexpressed subunits were synthesized in the batch-mode. Subsequently the SN fraction (**a**) and the MF fraction (**b**) were analyzed. An NTC was used as a negative control. Standard deviations were calculated from triplicate samples of two independent experiments (*n* = 6). (**c**) Morphological changes of CaCo2 cells exposed to the Hbl complex and an NTC volume equivalent.

The putative toxicity of the individual subunits as well as of two coexpressed subunits was evaluated. Individual subunits and two coexpressed subunits were compared to a coexpressed tripartite toxin complex. An NTC in a volume equivalent to the tripartite complex was analyzed in parallel. Three different concentrations were used for the SN and the MF fraction. At 0.5 nM the Hbl complex in the SN fraction showed cytotoxic activity, while the single subunits and two coexpressed subunits did not (Figure 7a). Analyzing the soluble subunits and two coexpressed subunits at 0.5, 1.5 and 2.5 nM revealed that with rising concentrations, the fluorescence intensity of L_2_ was on the same level as the Hbl complex, while coexpressed subunits L_1_ and L_2_ were even higher as the coexpressed tripartite toxin (Figure 7a), which suggests a pre-pore formation interacting with the cell membrane. The evaluation of the MF showed that no individual subunit and no coexpression of two subunits led to fluorescence counts as high as detected for the Hbl complex (Figure 7b). Strikingly, coexpressed B and L_2_ as well as L_1_ and L_2_ at 0.25 nM led to an increase in RLU but still the highest values were detected for the coexpressed Hbl. In order to assess these data, a morphological analysis of CaCo2 cells subjected to the Hbl complex, its single subunits and two coexpressed subunits was performed. Therefore, the SN fractions were administered to CaCo2 cells at a final concentration of 2.5 nM and the MF fractions were administered at 0.25 nM for 4 h in accordance to PI uptake assays. The Hbl complex perforated the cells leading to cell death whilst the NTC did not (Figure 7c). Strikingly, SN and MF both showed strong cytotoxic effects on CaCo2 cells. Single subunits and two coexpressed subunits did not induce cell death. Cells exposed to the SN fraction of coexpressed L_1_:L_2_ subunits showed a reduced confluency, suggesting a pre-pore formation attacking the cells (Figure S8).

## 3. Discussion

*Bacillus cereus* strains typically express a variety of toxic proteins among them complex enterotoxins, which cause foodborne diarrheal illness. Until today, no strain has been identified only expressing Hemolysin BL (Hbl). In general, strains that express Hbl also contain the second tripartite toxin Nhe [25,26]. To study a specific toxin, time-consuming purification steps are mandatory and need to be optimized in order to guarantee a pure toxin when working with bacterial cultures. Optimized purification procedures have been developed for the Hbl complex, but in the case of tripartite toxins the method has to be adapted and individually optimized for all three subunits [7,8]. As the coding sequences for the B, L_1_ and L_2_ subunits were identified in the 1990s [27,28], the specific toxin compounds could be overexpressed in vivo in bacterial strains [9,10,12,29]. Cell-free protein synthesis (CFPS) represents an efficient method to synthesize such toxic proteins. CFPS does not rely on the cell’s viability as a cell lysate is used which provides ideal prerequisites for the synthesis of toxic proteins [30]. This study describes the cell-free synthesis of the Hbl complex and its individual subunits for the first time. In addition to that, this is the first study to show the synthesis of Hbl in a eukaryotic system. Using a eukaryotic cell-free system resolves the need to purify the synthesized protein prior to functionality testing which speeds up the screening process. In our study, we show that the cell-free synthesis of each individual subunit as well as a coexpression of two and all three subunits of the Hbl tripartite enterotoxin is possible. In addition to these data, each individual subunit as well as the coexpressed ones were analyzed. Hbl proteins were probed in solution as well as when interacting with the microsomal vesicles present in the CHO lysate. Table S1 summarizes the data acquired for the hemolytic activity of Hbl. The hemolytic activity of Hbl could be characterized in versatile ways using a eukaryotic cell-free system. Coexpression of all three subunits as well as mixing synthesized individual subunits led to hemolytically active enterotoxin complexes. Using a eukaryotic system, we could reproduce previous findings such as the lytic activity when all three subunits are present but no lytic activity when a single subunit is absent. Further, a crescent zone phenomenon can be achieved when the subunits are in close proximity on the blood agar plates which was shown both in our study and in previous work (Figure 2 and Figure S1) [5]. 

As Hbl is a soluble protein, the majority of active protein is present in the soluble fraction as compared to the MF (Figure 2a). Prior studies stated that two structures, namely the LPS-Induced TNF-α Factor (LITAF) and the cell death inducing p53 target 1 (CDIP1), are needed for Hbl to target the cell [31]. These structures are generally expressed in tissues but are thought to play a role in endosomal protein degradation (the Human protein Atlas). A clear localization to the endoplasmic reticulum cannot be stated. This indicates that the microsomal vesicles used in this study do not harbor LITAF and CDIP1 which could lead to lower protein yields in the MF as no protein accumulation can take place. Even though the Hbl protein did not accumulate in a membranous surrounding leading to a lower protein yield in the MF, the microsomal vesicles in the CHO lysate are beneficial for CFPS. When a microsome depleted lysate was compared to a microsome enriched lysate as presented in Figure 3a, the overall protein yield was reduced without microsomal vesicles. This might be due to the fact that membrane bound ribosomes at the ER derived microsomes are still present and favor protein translation.

Nonetheless, our results indicated that Hbl might partly interact with the vesicles present in the CHO lysate. We analyzed the membrane association to ER-structures. Using a proteinase K digestion we were able to show that the B Component interacts with the ER-based microsomal vesicles present in our eukaryotic cell-free system whilst the other subunits did not (Figure 3c and Figure S3). These data add to previous findings that the B Component anchors to the cell and further allows for complex formation [3,4] showing that not only cell surfaces can be targeted but any lipid bilayer. Hemolytic activity of the MF was concentration dependent (Figure 2d). The MF fraction showed hemolytic activity that was not as intense as for the soluble protein (Figure 2c) which is likely caused by a concentration-dependent activity on blood agar plates. When studying Hbl subunit interactions our study assessed the different molar plasmid concentrations added to the synthesis as well as the different final protein ratios whilst prior work assessed the protein subunits expressed in bacterial cultures [4,6,10,12]. In general, both the coexpressed Hbl complex and the complex formed when subunits were mixed after the cell-free synthesis were hemolytically active (Figure 4). Studies describing the interaction of the different subunits stated that if the L_1_ subunit was available in excess to the other subunits, the hemolysis was inhibited [6] and pore-formation was slowed [12]. Our work showed lytic activity for all different combinations of molar plasmid ratios as well as molar protein ratios for the SN fraction, except the protein ratios 1:10:1 and 1:1:10 (B: L_2_:L_1_) (Figure 4 and Figure S5). This confirms that an excess of L_1_ acts inhibitory and it further indicates that also an excess of the L_2_ subunit over both the B and L_1_ subunit may impede hemolysis. However, when interpreting these data, it has to be considered that in this study the assessment of lytic activity is only a qualitative analysis and quantitative analyses using precise numbers of erythrocytes could predict the slightest hemolytic activities in the future. Further, an incubation on blood agar plates for 24 h might not be suitable to assess a slower pore-formation. An earlier study has shown that an excess of the B Component inhibits the lytic activity of L_1_ [6]. Within our study, the B Component did not limit the lytic activity at high concentrations. When adding the B Component in a membrane associated way, higher concentrations were needed to facilitate the lytic activity (Figure 4c). Prior studies indicated a sequential binding order of the B Component followed by the lytic component L_1_ and L_2_ at last [9]. Moreover, the formation of soluble B and L_1_ as well as L_1_ and L_2_ complexes could be verified by SPR, Dot Blot analysis and EIA [10]. The data presented in this study might suggest that the B Component was not fully capable to form pre-complexes with L_1_ or anchor L_1_-L_2_ soluble complexes to the erythrocytes in the MF fraction (Figure 4b,c). Further findings showed that when the binding component was added to erythrocytes before adding the lytic components, lysis occurred while an addition of the binding component after the lytic components did not induce lysis [5]. When subunits were fused after cell-free synthesis by an incubation step on ice, no inhibition of the lytic activity could be detected regardless of the combination order of Hbl proteins (Figure 5). Thus, these data indicate that without the presence of erythrocytes the complex formation is not dependent on the sequential binding order. Previous work has shown that the pore-formation of Hbl is similar to Hemolysin E from *E. coli* including a putative oligomerization of the B Component into hepta- and octamers [11]. Another study has proven the formation of complexes between the lytic component L_1_ and the binding component as well as between both lytic components in solution [10].

The detailed analysis of membrane integrity is best suited to characterize the mode of action of a pore-forming protein and therefore might detect pre-pore complexes. A cell-culture based propidium iodide uptake assay indicated cytotoxicity of the Hbl complex (Figure 6) in accordance to previous data [12]. The data acquired in this study are summarized in Table S2. Jessberger et al., showed a concentration dependent cytotoxic activity of Hbl enterotoxin on Vero cells similar to the results presented here. These previous findings also showed that a 50% inhibition rate of the cell viability was present at 0.3 nM Hbl. In comparison to that, cell-free synthesized Hbl showed initial cytotoxic activity at a concentration of 0.1 nM but could specifically be detected at 0.25 nM in the SN fraction (Figure 6a), depicting a similar activity pattern of cell-free synthesized Hbl. Comparing the SN and MF fraction, toxic effects of the MF could already be depicted at concentrations of 0.05 nM (Figure 6b) as the NTC background measurement presented less intense signals. In contrast to our data, Jessberger et al., further showed that a PI influx was only observed when all three subunits were present during the incubation time on the cells and at an approximate concentration of 3.75 nM no toxic effects for single subunits could be detected [12]. Our study showed that increasing concentrations of expressed subunits, especially coexpressed subunits, showed high background signals (Figure 7). These data also depict that the PI uptake assay is applicable to assess cell-free synthesized proteins when lower protein concentrations are applied. At higher target protein concentrations, and when higher volumes of the cell-free reaction were used, background noise could be detected, and further methods should be used such as the MTT viability assay. These background signals might originate from unspecific interactions with the lysate as well as nucleic acids present in the sample mixture. As depicted in Figure 6, NTC values were higher when using the SN fraction. The overall protein yield was higher in the SN as compared to the MF which led to the use of much higher concentrations of SN protein in the various assays used in this study. This might have led to higher background noise from the lysate as well as the other components added to the synthesis because SN contains a higher fraction of endogenous proteins compared to MF. When assessing the cytotoxic effect of the SN and MF fraction on CaCo2 cells, apoptotic effects were clearly seen for both fractions (Figure 7) whilst the RLU detected by the PI uptake assay showed higher values for the SN samples. The NTC though did not induce morphological changes, neither when the SN nor when the MF was applied, indicating that the lysate background itself was not cytotoxic. Despite the drawbacks when using the SN fraction, these data demonstrate that the PI uptake assay is an eligible tool to assess the membrane perforation effect. Using low concentrations of cell-free synthesized protein, membrane perforating effects could be detected. This underlines the fact that the PI uptake assay in combination with CFPS can be used for toxicity screenings of pore-forming proteins. The PI uptake assay showed higher RLU values at high concentration for coexpressed L_1_-L_2_ subunits. Strikingly, they did not show hemolytic activity (Figure 2). This might be due to the effect that the sample used in this study is not a purified sample. A morphological analysis of CaCo2 cells was performed to assess potential background noise. The data obtained indicate that only the entire Hbl complex induced cell death. In contrast, the single subunits and two coexpressed subunits did not clearly affect the CaCo2 cell viability based on morphological analysis. However, our data also showed a slightly reduced cell confluency when cells were subjected to L_1_:L_2_ compared to cells subjected to the NTC to untreated cells (Figure 7c and Figure S8). These data might indicate a pre-pore-formation of L1 and L2 in a soluble manner interacting with the cell membrane and thereby facilitating the entry of PI into the cell. Such a soluble pre-pore-complex-formation has already been previously described by Tausch et al., 2017, suggesting that cell-free protein synthesis can be applied for the analysis of pre-pore complexes [10]. A pre-pore formation is often detected in the mechanism of action of pore-forming proteins such as Perfringolysin, ClyA or even Nhe from *B. cereus* [32,33,34]. After binding to specific receptors on the cell surface, the pore-forming protein enriches at the targeted cell. With the increased concentration of the protein of interest multimerization and pre-pore-complexes occur. Even though L_1_ and L_2_ did not show cell binding properties in previous work [9], Tausch et al., 2017 was the only study yet assessing the detection of the L_1_-L_2_ complex [10]. The findings in this study indicate that Hbl-subunits synthesized in a cell-free manner could -assemble to the whole complex or sub-complexes in a soluble as well as a putative membrane associated surrounding. The putative assembly of a pre-pore complex thus has to be further evaluated.

The data acquired in this study qualify CFPS as a promising technology to synthesize and analyze multicomponent proteins in a soluble and membrane associated way. Our results demonstrate that eukaryotic cell-free systems enable the synthesis of a functionally active tripartite Hbl complex via coexpression and when mixing Hbl proteins immediately after their synthesis. In future studies, CFPS might be a valuable tool to study the interaction of Hbl with different toxins from *Bacillus cereus* such as sphingomyelinases and phosphatidylcholine phospholipases C. In this context, each individual toxin could be synthesized and mixed after cell-free synthesis to study the functional activity of differently combined subunits to resemble the natural conditions. Further in this context, CFPS might help to further elucidate the structural assembly of the Hbl complex. Summarizing, these data depict the significance of CFPS for the synthesis and functional characterization of both soluble and membranous cytotoxic proteins.

## 4. Material and Methods

### 4.1. Cell-Free Protein Synthesis

Cell-free synthesis reactions using translationally active lysate derived from Chinese hamster ovary cells (CHO-K1) were performed as described previously [24,35,36]. Shortly, CHO-K1 cells were grown in suspension cultures at 37 °C in controlled fermenters. Cells were cultivated in Power CHO-2 serum free medium (Lonza, Basel, Switzerland) and were harvested at a density of 2 × 10^6^ cells/mL. After a centrifugation at 200× *g* for 10 min, the cell pellet was washed with 40 mM HEPES-KOH (pH 7.5), 100 mM NaOAc, and 4 mM DTT. Subsequently, cells were disrupted through a micro-emulsifying process using a 20-gauge needle. After a 10 min centrifugation at 10,000× *g*, the supernatant was applied to a size-exclusion chromatography column (Sephadex G-25, GE Healthcare, Munich, Germany). Endogenous mRNA was digested by a micrococcal nuclease (S7) treatment for 2 min. The reaction was stopped by adding 6.7 mM EGTA. Lysates were stored at −80 °C until further use. Plasmids encoding each individual subunit of the Hbl enterotoxin were designed according to Brödel et al., 2013 including the internal ribosome entry site (IRES) of the cricket paralysis virus (CrPV) genome to allow for a cap independent translation initiation [37]. Genes encoding each subunit were obtained by de novo gene synthesis (Biocat GmbH, Heidelberg, Germany). Gene sequences derived from *Bacillus cereus* strain ATCC 14,579 were used: AJ237785.1:13463–14590 for the B Component, AJ237785.1:10826–12169 for L_1_ and AJ237785.1:12206–13426 for L_2_. The coding sequence of the native secretory signal peptide was removed so that only the mature protein was present after cell-free synthesis. All sequences were cloned into the pUC57-1.8K vector backbone and the plasmids were directly used as a template in cell-free reactions. These plasmids were either used separately, in combinations of two plasmids or in a coexpression of all three plasmids to assemble the tripartite toxin. Further, a no template control (NTC) consisting of a translation mixture without any DNA template was used as a background control.

### 4.2. Batch-Based Reactions

Protein synthesis was conducted in coupled transcription/translation reactions in a final volume of 25 to 80 μL. Batch-based reactions were incubated in a thermomixer (Eppendorf, Wesseling-Berzdorf, Germany) for 3 h at 30 °C and 500 RPM. Cell-free synthesis reactions were composed of 40% (*v*/*v*) translationally active CHO lysate supplemented with HEPES-KOH (pH 7.6, f.c. 30 mM, Carl Roth GmbH, Karlsruhe, Germany), sodium acetate (f.c. 100 mM, Merck, Darmstadt, Germany), Mg(OAc)_2_ (f.c. 3.9 mM, Merck), KOAc (f.c. 150 mM, Merck), amino acids (complete 100 μM, Merck), spermidin (f.c. 0.25 mM; Roche, Grenzach, Germany), Dithiothreitol (DTT, 2.5 mM, Life technologies GmbH, Carlsbad, USA ) and energy regenerating components including creatine phosphokinase (f.c. 0.1 mg/mL, Roche), creatine phosphate (20 mM, Roche), ATP (1.75 mM, Roche) and GTP (0.3 mM, Roche). To allow for DNA transcription during cell-free protein synthesis, 1 U/μL T7 RNA polymerase, 0.3 mM of UTP (Roche) and CTP (Roche) and 0.1 mM of the cap analogue m7G(ppp)G (Prof. Edward Darzynkiewicz, Warsaw University, Warsaw, Poland) were added to the reaction. Additionally, PolyG primer (f.c. 12 µM, IBA, Göttingen, Germany) was supplemented. For further analyses including autoradiography and liquid scintillation counting, cell-free protein synthesis reactions were supplemented with radioactive ^14^C-leucine (f.c. 50 μM, specific radioactivity 66.67 dpm/pmol, Perkin Elmer, Baesweiler, Germany).

After 3 h of incubation the crude translation mixture (TM) was centrifuged (16,000× *g*, 10 min, 4 °C) resulting in the supernatant (SN), containing the soluble subunits and the pelleted microsomes containing putative membrane bound subunits. The pellet was resuspended in phosphate buffered saline (PBS) resulting in the microsomal fraction (MF).

### 4.3. Analysis of Radio-Labeled Proteins

Total protein yields of cell-free synthesized proteins were determined by incorporation of ^14^C-leucine and subsequent precipitation by hot trichloro acetic acid (TCA, Carl Roth GmbH). Briefly, 3 µL aliquots of the fraction of interest (TM, SN or MF) were mixed with 3 mL of 10% TCA/2% casein hydrolysate (Carl Roth GmbH) solution and incubated at 80 °C for 15 min. After a 30 min incubation on ice, ^14^C-labeled proteins were transferred to membrane filters (VWR) using a vacuum filtration system (Hoefer, Holliston, USA). Filters were washed twice with 5% TCA to remove non-incorporated ^14^C-leucine and dried with acetone. Dried filters were placed in 3 mL scintillation cocktail, incubated for at least 1 h and measured by liquid scintillation counting using the LS6500 Multi-Purpose scintillation counter (Beckman Coulter, Germany). The total protein yield of a single protein was calculated according to the following equations:

(1) $$\mathrm{Protein}\;\mathrm{yield}\;\left[\frac{µg}{\mathrm{mL}}\right]=\frac{\mathrm{scintillation}\;\mathrm{counts}\left[\frac{\mathrm{dpm}}{\mathrm{mL}}\right]\times \;\mathrm{molecular}\;\mathrm{weight}\left[\frac{µg}{\mathrm{pmol}}\right]}{\mathrm{specific}\;\mathrm{radioactivity}\;\left(A_{\mathrm{spec}}\right)\;\left[\frac{\mathrm{dpm}}{\mathrm{pmol}}\right]\times \;\mathrm{number}\;\mathrm{of}\;\mathrm{leucines}\;}$$

(2) $$A_{\mathrm{spec}}\;\left[\frac{\mathrm{dpm}}{\mathrm{pmol}}\right]=\frac{\mathrm{stock}\;\mathrm{concentration}\;\mathrm{of}\;14C\;\mathrm{leucine}\;\left[µM\right]\times {\;A}_{\mathrm{spec}}\;\mathrm{of}\;14C\;\mathrm{leucine}\;\mathrm{stock}\;\left[\frac{\mathrm{dpm}}{\mathrm{pmol}}\right]}{\mathrm{Total}\;\mathrm{concentration}\;\mathrm{of}\;\mathrm{leucine}\;\left[µM\right]}$$

Total protein yields of de novo synthesized toxin subunits were analyzed using liquid scintillation counting. The total protein yield of coexpressed subunits was estimated using the sum of the molecular weight and the sum of the number of leucines of all expressed subunits.

For qualitative analysis, 3 µL aliquots of the fraction of interest were precipitated in cold acetone (Carl Roth GmbH) and handled as described previously [38]. Sodium dodecyl sulfate polyacrylamide gel electrophoresis (SDS-PAGE) using precast gels (NuPAGE, 10% Bis-Tris, Life technologies) and self-prepared 10% gels using SureCast resolving and stacking buffer (Thermo Fisher Scientific, Rockford, USA ) was performed to confirm the molecular mass of radio-labeled, cell-free synthesized proteins. Precast gels were run at 185 V for 35 min while self-prepared gels were run at 150 V for 55 min. At last, gels were dried at 70 °C (Unigeldryer 3545D, Uniequip, Planegg, Germany), placed on a phosphor screen (GE Healthcare) and radioactively labeled proteins were visualized using a Typhoon Trio and variable mode imager (GE Healthcare).

### 4.4. Proteinase K Digestion

The protein digestion assay was performed in a final volume of 10 µL. A 5 µL aliquot of the MF was incubated with PBS containing the Proteinase K (NEB) at a final concentration of 1 ng/µL for 30 min on ice. Afterwards the mixture was precipitated with acetone and qualitative analysis was performed as described above.

### 4.5. Hemolytic Activity Assessment

Hemolytic activity of cell-free produced Hbl enterotoxin was assessed using precast blood agar plates containing 5% sheep blood (VWR). A total volume of 10 µL of de novo synthesized toxin was directly spotted onto the blood agar plate. TM, SN and MF fractions of the cell-free reactions were analyzed. To suppress microbial growth, erythromycin and gentamycin (f.c. 50 µg/mL) were added to each sample. After 24 h of incubation at 37 °C, hemolytic zones were visualized and documented. Triton-X 100 detergent solution was used as a positive control. To analyze defined toxin concentrations, protein yields were initially determined by TCA-precipitation (described in the section entitled “Analysis of Radio-Labeled Proteins”). Subsequently, defined concentrations were calculated, and corresponding volumes were spotted onto the blood agar plate. Experiments were repeated three times.

### 4.6. Membrane Perforation Studies

In order to assess the insertion of the pore-forming Hbl toxin into the cell membrane, a propidium iodide uptake assay was performed. Therefore, the epithelial colorectal adenocarcinoma cell line CaCo2 (ATCC) was cultured in minimum essential medium (MEM) supplemented with 20% fetal calf serum (Merck, Biochrom GmbH, Berlin, Germany), 1% non-essential amino acids (Merck, Biochrom GmbH) and 1% penicillin/streptomycin (Merck, Biochrom GmbH). In this study, 10,000 CaCo2 cells in 150 µL medium were seeded into 96-well microplates. Cells were allowed to adhere for 24 h and subsequently 20 µL of either the SN or the MF fraction of the cell-free synthesized tripartite enterotoxin were added to each well. Each sample was analyzed in triplicates, if not stated otherwise. As negative controls untreated cells and an NTC synthesized in a batch reaction were used. The latter was diluted and treated in the same manner as the proteinaceous toxin used in the assay and applied in a volume equivalent to the toxin. Toxin concentrations of the radio-labeled SN and MF fractions were measured by TCA-precipitation (see above) and, consequently, assumed for non-labeled fractions as both reactions were prepared in parallel. Varying concentrations from 0.01 nM to 2.5 nM were studied. Additionally, 30 µL of a propidium iodide (MP Biomedicals, Irvine, USA, purchased from VWR, Darmstadt, Germany) solution was added to each well. A final propidium iodide concentration of 10 µg/mL was used. Cells were incubated for 4 h in a cell incubator at 37 °C with 5% CO_2_. If the membrane of the cell was disrupted by the enterotoxin Hbl, propidium iodide could enter the cell and bind to the DNA. The emission of the propidium iodide was measured at 616 nm using the Mithras LB 943 (Berthold Technologies, Bad Wildbad, Germany) in relative light units (RLU). The mean of each triplicate measurement was calculated and subtracted by the mean triplicate measurement of medium treated cells.

### 4.7. Morphological Changes of CaCo2 Cells

Morphological changes of CaCo2 cells were analyzed to assess the toxicity of the Hbl enterotoxin. Therefore, cells were seeded into 48 well plates at 15,000 cells/well in 450 µL. Cells were incubated for 24 h to allow for adhesion. Afterwards, the cell-free synthesized toxin was diluted in cell culture medium to the desired concentration and a final volume of 50 µL. These 50 µL were added to the individual wells. Further, 4 h later morphological changes were documented using a light microscope. Phase contrast micrographs were captured with a CCD camera (Nikon, Melville, USA).

## Data Availability

All relevant data are within the paper and its Supporting Information Files.

## Supplementary Materials

The following supplementary material is available online at https://www.mdpi.com/article/10.3390/toxins13110807/s1: Figure S1: Hemolytic assessment of Hbl subunits, Figure S2: hemolytic assessment of Hbl complex, Figure S3: proteinase K digestion, Figure S4: Uncropped 5% sheep blood agar plates from Figure 3, Figure S5: Analysis of subunit interaction at different molar ratios, Figure S6: Uncropped 5% sheep blood agar plates from Figure 4, Figure S7: Uncropped 5% sheep blood agar plates from Figure 5, Figure S8: Morphological analysis of CaCo2 cells. Table S1: Summary of acquired data: hemolytic activity of Hbl. Table S2: Summary of acquired data: cytotoxic activity of Hbl.
