# Supplementary material for: The Pore-Forming Hemolysin BL Enterotoxin from Bacillus cereus: Subunit Interactions in Cell-Free Systems
Source: Toxins (Basel). 2021 Nov 15;13(11):807. doi: 10.3390/toxins13110807 (PMC8623112; doi:10.3390/toxins13110807)
Supplement: Supplementary file 1 [file toxins-13-00807-s001.zip › toxins-1439190 supplementary.pdf]

Supplementary Materials: The Pore-Forming Hemolysin BL Enterotoxin from *Bacillus cereus*: Subunit Interactions in Cell-Free Systems

Franziska Ramm, Marlitt Stech, Anne Zemella, Hendrik Frentzel and Stefan Kubick

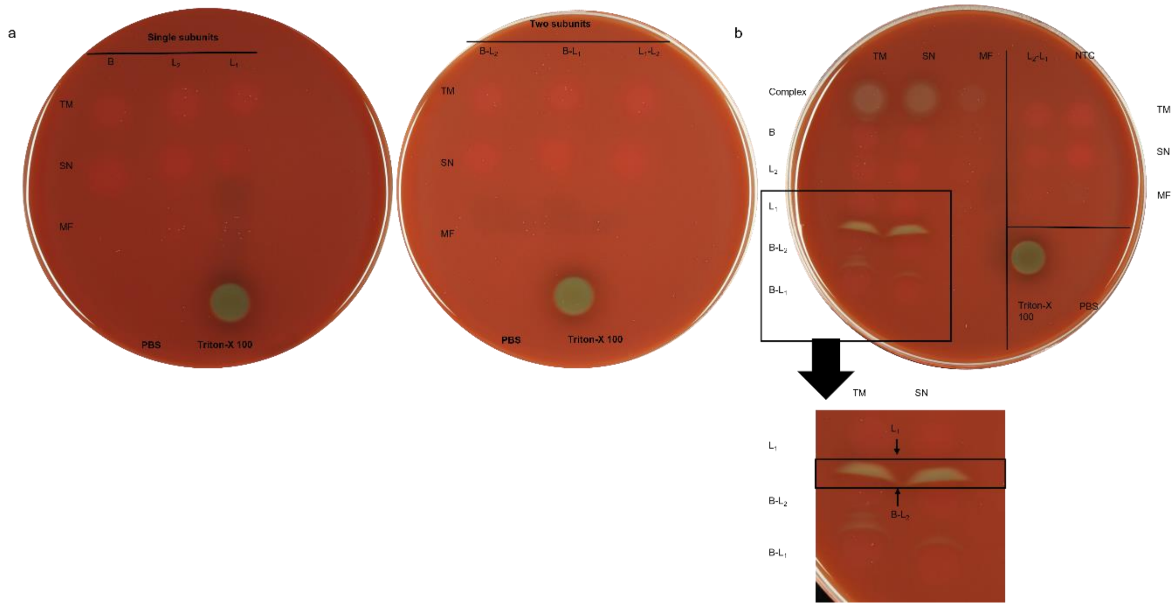

Figure S1. Hemolytic assessment of Hbl subunits. Hbl subunits B, L_1 and L_2 were synthesized in CHO lysate either separately or in a coexpression of either two or three subunits. (**a**) Uncropped 5% sheep blood agar plates from Figure 2 c. (**b**) Hemolytic activity of the single subunits and two coexpressed subunits was assessed on one single 5% sheep blood agar plate. Arrows indicating which subunit diffused in which direction.

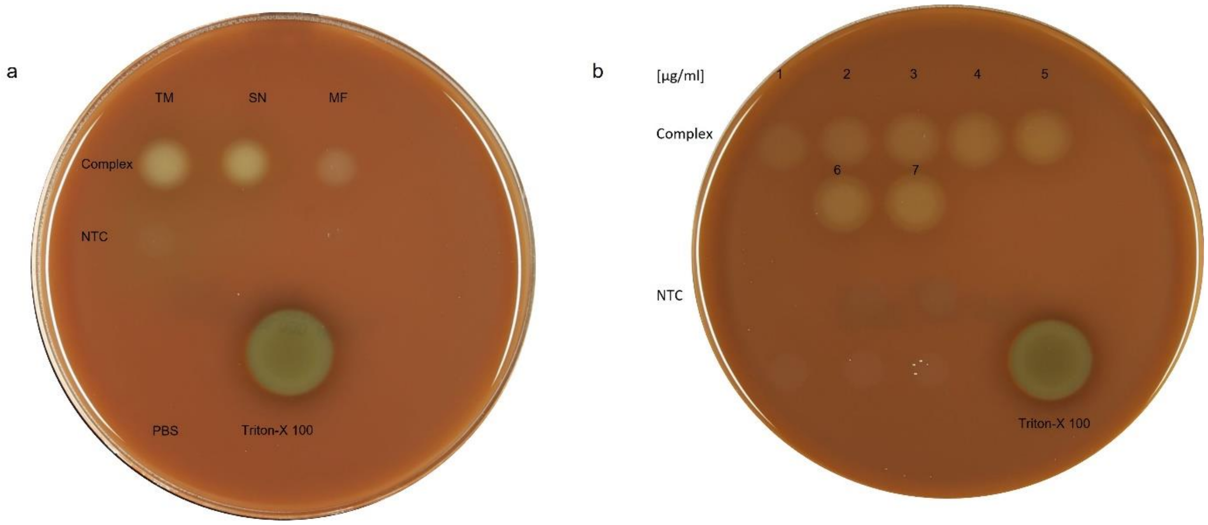

Figure S2. Hemolytic assessment of Hbl complex. Hbl subunits B, L_1 and L_2 were synthesized in CHO lysate in a coexpression of all three subunits. (**a**) Uncropped 5% sheep blood agar plate from Figure 2 d. (**b**) Hemolytic activity of the coexpressed tripartite Hbl enterotoxin assessed on 5% sheep blood agar plate in a concentration range from 1 to 7 µg/ml.

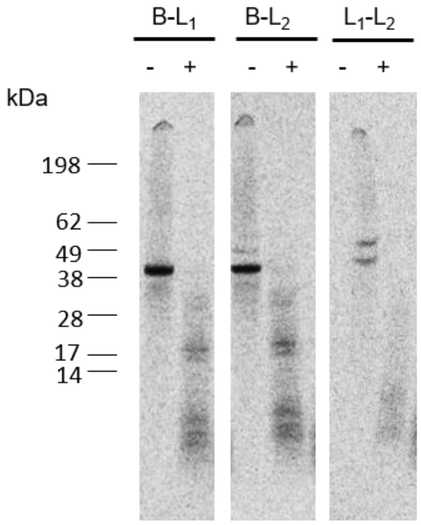

Figure S3. Proteinase K digestion. Autoradiograph showing ^{14}C-leucine labeled coexpressed subunits when synthesized using molar plasmid ratio of 1:1 before (−) and after (+) a proteinase K digestion.

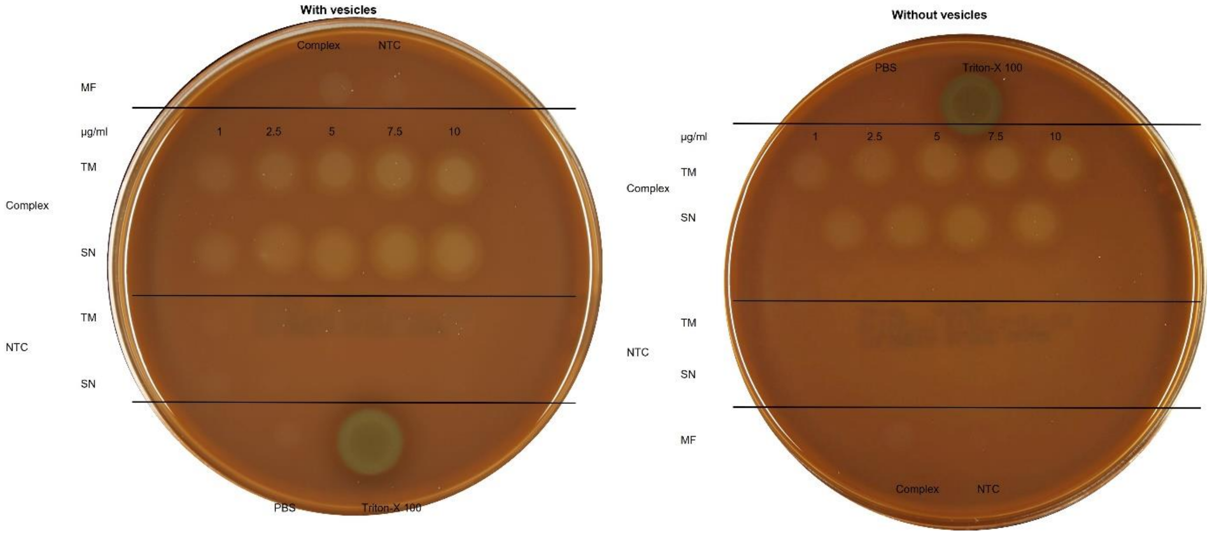

Figure S4. Uncropped 5% sheep blood agar plates from Figure 3.

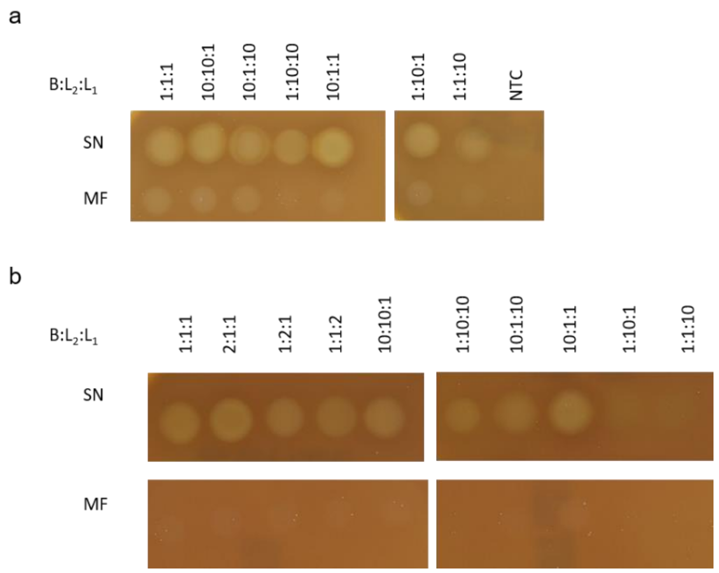

Figure S5. Analysis of subunit interaction at different molar ratios. Hemolytic activity was assessed on 5% sheep blood agar plates. (**a**) Hbl subunits B, L_2 and L_1 were coexpressed in CHO lysate using different molar plasmid ratios. (**b**) Hbl subunits were expressed separately in CHO lysate. Subsequently, fractions of each subunit in SN and MF were mixed in different molar protein ratios.

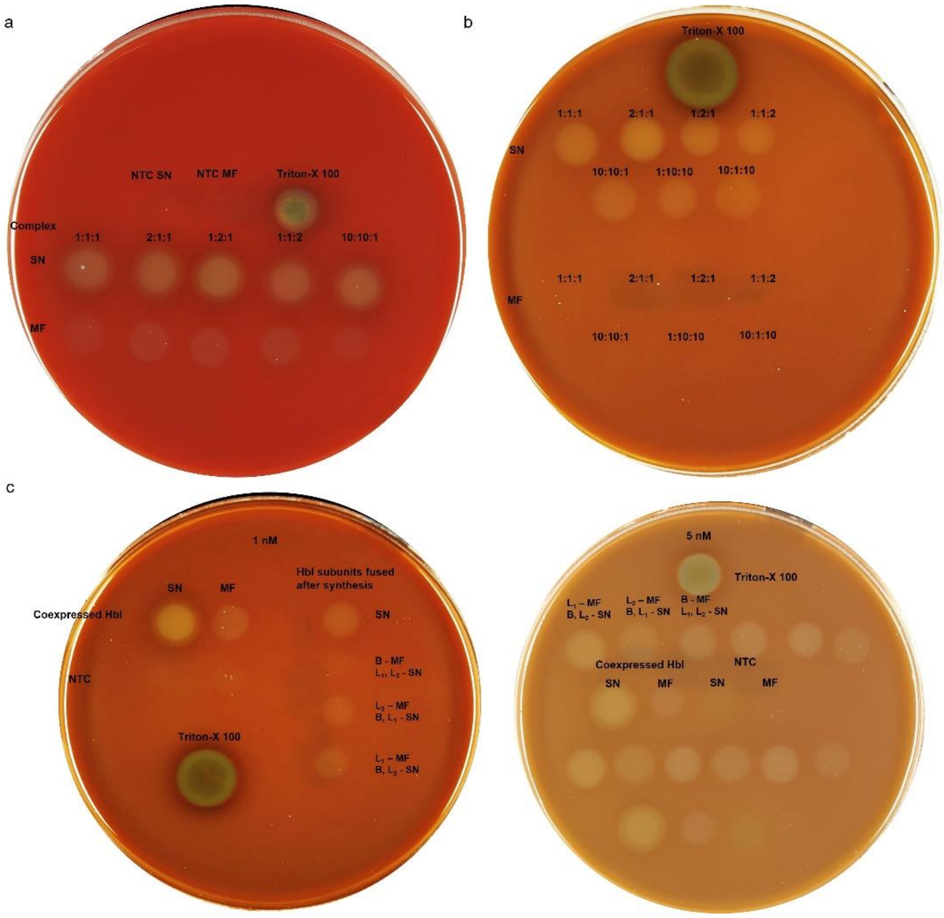

Figure S6. Uncropped 5% sheep blood agar plates from Figure 4**.**

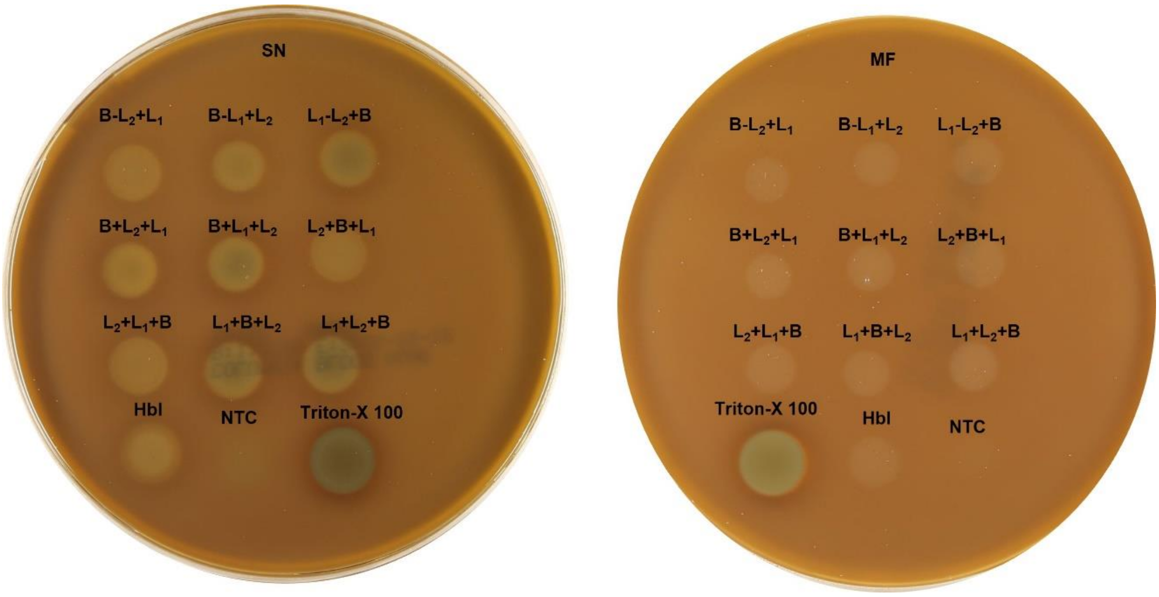

Figure S7. Uncropped 5% sheep blood agar plates from Figure 5.

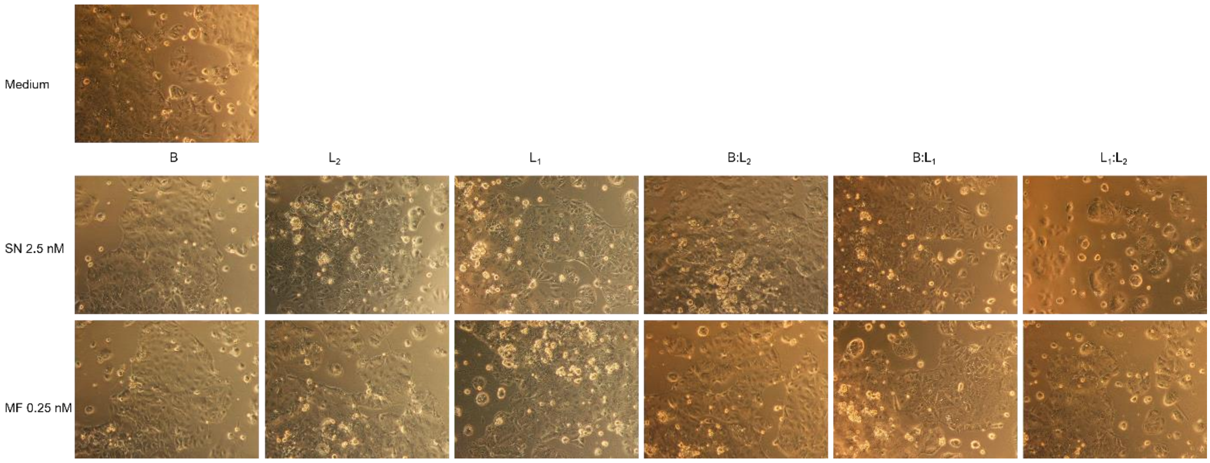

Figure S8. Morphological analysis of CaCo2 cells. Hbl single subunits or two coexpressed subunits were synthesized in a CHO lysate and added to CaCo2 cells. The SN fraction was applied at 2.5 nM while the MF fraction was applied at 0.25 nM. 4 h after incubation morphological changes were analyzed using a light microscope. Phase contrast micrographs were captured with a CCD camera (Nikon).

Table S1. Summary of acquired Data: Hemolytic activity of Hbl.
✓ = present, - = not present, n.d.a. = no data available.

Characteristic	Fraction	B	L_1	L_2	Hbl Complex		
					Combination ($B:L_2:L_1$)	Coexpression	Single subunit expression and fusion
**Hemolytic activity**	SN	-	-	-	1:1:1	✓	✓
					2:1:1	✓	✓
					1:2:1	✓	✓
					1:1:2	✓	✓
					10:10:1	✓	✓
					10:1:10	✓	✓
					1:10:10	✓	✓
					10:1:1	✓	✓
					1:10:1	✓	-
					1:1:10	weak	-
	MF	-	-	-	1:1:1	✓	(weak)
					2:1:1	Concentration dependent	(weak)
					1:2:1	Concentration dependent	(weak)
					1:1:2	Concentration dependent	(weak)
					10:10:1	✓	(weak)
					10:1:10	✓	-
					1:10:10	weak	-
					10:1:1	weak	weak
					1:10:1	✓	-
					1:1:10	-	-
**Crescent formation**	SN		✓			n.d.a.	
	MF		-			n.d.a.	

Table S2. Summary of acquired Data: Cytotoxic activity of Hbl.
✓ = present, - = not present, n.d.a. = no data available.

Characteristic	Fraction	B	L_1	L_2	Hbl Complex		
					Combination ($B:L_2:L_1$)	Coexpression	Single subunit expression and fusion
**Membrane embedding**	MF	✓	-	-		✓	n.d.a.
**Membrane integrity**	SN	Interaction with cell membrane detected			1:1:1	✓	n.d.a.
	MF	-	-	-	1:1:1	✓	n.d.a.
**Cytotoxicity**	SN	-	-	-	1:1:1	✓	n.d.a
	MF	-	-	-	1:1:1	✓	n.d.a
